# Downsides to the empathic brain? A review of neural correlates of empathy in major depressive disorder

**DOI:** 10.3389/fnhum.2024.1456570

**Published:** 2024-08-15

**Authors:** Dahna Choi, Katharina Förster, Nina Alexander, Philipp Kanske

**Affiliations:** ^1^Clinical Psychology and Behavioral Neuroscience, Department of Psychology, Institute of Clinical Psychology and Psychotherapy, Technische Universität Dresden, Dresden, Germany; ^2^Department of Psychiatry and Psychotherapy, University of Marburg, Marburg, Germany; ^3^Center for Mind, Brain and Behavior, University of Marburg, Marburg, Germany

**Keywords:** major depressive disorder, social cognition, empathy, neural correlates, fMRI

## Abstract

Empathy as one of the basic prerequisites for successful social interactions seems to be aberrant in individuals with major depressive disorder (MDD). Although understanding empathic impairments in MDD is crucial considering the frequently reported social skill deficits in patients, the current state of research is still inconclusive, pointing to both elevated and impaired levels of empathy. In this review, we extend previous reports of MDD-related aberrations in self-reported and behavioral empathy by shedding light on the neural correlates of empathy in MDD. Study findings indicate a complex and potentially state-dependent association, comprising both elevated and lower neural activity in empathy-related brain regions such as the inferior frontal gyri, bilateral anterior insulae, and cingulate areas. Predominantly, lower activity in these areas seems to be induced by antidepressant treatment or remission, with accompanying behavioral results indicating a reduced negativity-bias in empathic processing compared to acute states of MDD. We propose a preliminary model of empathy development throughout the course of the disorder, comprising initially elevated levels of empathy and a somewhat detached and lower empathic responding during the further progression of the disorder or post-treatment. The seemingly multifaceted nature of the association between empathy and MDD requires further exploration in future multimodal and longitudinal studies. The study of neural correlates of empathy in MDD should prospectively be enlarged by including further socio-affective and -cognitive capacities in MDD and related mental disorders.

## 1 Introduction

Being empathic toward other people is one of the basic prerequisites for successful social interactions. This is reflected in various empirical findings on empathy being linked to positive intra- and interpersonal outcomes such as relationship satisfaction and prosocial behavior (Bailey et al., 2008; Chow et al., 2013; Lehmann et al., 2022). Accordingly, lower levels of empathy show associations with psychopathological conditions, for example schizophrenia or autism spectrum disorders (Bonfils et al., 2016; Harmsen, 2019).

Emerging evidence however also points to high levels of empathy not being unconditionally adaptive: Elevated empathy at the other end of the spectrum has also been suggested to be associated with negative mental health outcomes, potentially entailing a heightened sensitivity toward negative emotions (Chikovani et al., 2015; Green et al., 2018) and an exaggerated feeling of responsibility for another's suffering (Eisenberg et al., 2024). Although withdrawing from social situations as a coping strategy might alleviate one's discomfort in a particular moment, it yields the risk of social isolation in the long run (Buruck et al., 2014; Kim and Han, 2018).

In line with these assumptions, major depressive disorder (MDD) has been discussed to be associated with and triggered by an accumulation of negative affect resulting from empathizing with others' negative emotions (Ding et al., 2023). On the contrary, other researchers suggest that patients with MDD show reduced empathy compared to healthy control participants (HCs), supposedly due to a limited affective range and affective responsiveness (Field et al., 2009).

Either way, in line with the notion of empathy to be a “double-edged sword” (Russell and Brickell, 2015) or a “risky strength” (Tone and Tully, 2014), aberrations in the sense of either an elevation or reduction of empathy yield the potential of being a relevant mechanism underlying MDD-related difficulties in social interactions (Kupferberg et al., 2016). While MDD could result from elevated empathy—mediated by heightened distress by other's negative emotions—, it could also be triggered by reduced empathy that might interfere with an individual's social functioning and maintenance of social relationships. Understanding the relevance of empathic impairments in MDD is crucial when considering that social skill deficits are thought to contribute to the chronification of the disorder due to a loss of positive social reinforcement (Libet and Lewinsohn, 1973).

In contrast to previous studies having predominantly considered self-report measures of empathy that are prone to be affected by MDD-related cognitive biases (Schreiter et al., 2013; Kupferberg et al., 2016; Yan et al., 2021), we review studies on the neural correlates of empathy in association with MDD. We thereby focus on affective empathy, which we define as the sharing of another person's emotions, yielding an isomorphic affective state in the observer (De Vignemont and Singer, 2006). Such empathic affect sharing paves the way for differential functional outcomes: While it can result in adaptive, caring responses toward another person—also termed compassion—, it can also trigger empathic distress as a more self-oriented and aversive reaction (Singer and Klimecki, 2014). Affective empathy is to be distinguished from more cognitive aspects of empathy or Theory of Mind, comprising the ability to infer and reason about others' beliefs, thoughts, or emotions (Frith and Frith, 2005). Although affective and cognitive processes are likely to co-occur in naturalistic social situations, considering affective or cognitive empathy separately is required as a first step toward understanding their interplay with MDD. The differentiability between affective and cognitive routes of understanding others additionally shows in their neural distinctiveness: The bilateral anterior insulae (AI), inferior frontal gyri (IFG), and cingulate areas show associations with affective empathy, whilst brain areas related to cognitive empathy comprise the medial prefrontal cortex, middle temporal gyrus, and precuneus (Lamm et al., 2011; Kanske, 2018; Schurz et al., 2021).

Here, we summarize findings on aberrant empathy-related neural correlates in MDD patients at different disorder states and review changes after remission and treatment. Based on these findings, we propose a preliminary model of a state-dependent associational shift between empathy and MDD, which describes elevated levels of empathy before and during acute MDD and reduced empathy after remission and antidepressant treatment. Since socio-affective and -cognitive processes might not be readily tangible to the social agents, by elucidating the underlying neural mechanisms, we aim to provide new insights into the complex interplay between empathy and MDD.

## 2 Neural correlates of aberrant empathy at different states of MDD

Two studies on empathy for pain provided first indications on aberrations in empathy-related neural activity at different states of MDD. In Fujino et al.'s (2014) study, individuals with acute MDD and HCs were presented with videos showing painful and non-painful situations and were subsequently instructed to rate the pain intensity of the videos. Analyses revealed individuals with MDD to show less task-related neural activity in the left middle cingulate cortex (MCC) and somatosensory-related cortices—namely, the supramarginal gyrus and postcentral gyrus—, but elevated activity in the left IFG. On a self-report level, MDD patients indicated lower subjective pain ratings compared to HCs.

Based on prior studies on the MCC's and somatosensory-related cortices' functional implications and in accordance with lower self-reported pain ratings, the authors suggested inhibited activity in those brain regions to reflect reduced empathic processing in patients with MDD (Jackson et al., 2005). In contrast, considering the IFG's association with the regulation of negative emotions (Johnstone et al., 2007) and self-reported empathic distress (Saarela et al., 2006), elevated IFG activity might add to previous indications on higher empathic distress in MDD (Schneider et al., 2012).

While in Fujino et al.'s (2014) study, no inferences can be made on the associations between these neural aberrations and participants' disorder state, insights into the latter are provided in Rütgen et al.'s study from 2021: In an fMRI task, patients with acute MDD, remitted MDD patients, and HCs watched videos showing targets, alleged tinnitus patients, undergoing a painful noise treatment. Participants were then asked to rate the degree of unpleasantness that they thought the target person would feel (target unpleasantness rating), and the degree of unpleasantness for themselves when empathizing (self-experienced unpleasantness rating). fMRI analyses revealed that remitted MDD patients showed elevated activity in the right temporoparietal junction (TPJ) compared to patients with acute MDD and HCs. In the same group contrasts, remitted patients showed lower activity in areas associated with the processing of emotions and emotional facial expressions, such as the left visual association cortex, bilateral amygdalae, and the left AI. On a self-report level, remitted patients indicated higher ratings of target unpleasantness ratings compared to patients with acute MDD and HCs, while self-unpleasantness ratings were similar across all groups.

The authors interpret their findings as reflecting remitted MDD patients to cognitively anticipate more pain for the other and to show a higher self-other distinction reflected in elevated TPJ activity (Quesque and Brass, 2019; Borja Jimenez et al., 2020). Importantly, not only the stratification of the investigated sample based on disorder state but also the simultaneous assessment of the affective and cognitive processing of another's pain—measured in self-experienced and target unpleasantness ratings, respectively—provide a differential view on the association between empathy and MDD.

Although interpretations are preliminary, these studies indicate fluctuations in the association between empathy and MDD depending on the current disorder state. Elevated TPJ activity in remitted MDD might be reflective of elevated self-other distinction and higher cognitive empathic processing, which has meta-analytically shown to be one of the TPJ's major functional associations (Krall et al., 2015). Potentially, this might pose a learned compensatory mechanism counteracting elevated empathic distress at acute states of MDD. The generalizability of these findings on empathy for mere pain exposure to natural empathy-evoking situations as well as their longer-term implications require further investigation in future studies.

## 3 Treatment effects on neural correlates of empathy in MDD

Further studies on neural correlates of empathy in MDD provide important indications of a mediating role of empathy in mechanisms underlying symptom improvement after antidepressant treatment. In a study on empathy for pain from Rütgen et al. (2019) in which the same empathy for pain task was conducted as described above (Rütgen et al., 2021), HCs and patients with acute MDD performed the fMRI task before and 3 months after patients underwent antidepressant treatment.

Whilst no pre-treatment behavioral nor neural group differences were evident, after antidepressant treatment, MDD patients showed decreased neural activity in bilateral AI and the anterior MCC, as well as reduced self-experienced unpleasantness ratings compared to their pre-treatment responses and compared to HCs. Lower self-reported unpleasantness ratings in MDD patients moreover correlated with symptom improvement after treatment. These findings provide important indications of reduced affective processing of negative social information induced by antidepressant treatment, which is reflected in lower activity in empathy-related brain regions (Rütgen et al., 2019).

Although these interpretations are preliminary due to the data being correlational, further support is provided in two deep-brain stimulation (DBS) studies on patients with treatment-resistant MDD. In Merkl et al.'s (2016) study, oscillatory response patterns during the presentation of an empathy task (Dziobek et al., 2011) and the modulation of behavioral responses after 6 months of DBS were investigated. Patients underwent DBS in the subgenual anterior cingulate cortex (sgACC), as the sgACC has been shown to be a promising target for DBS in treatment-resistant MDD (Mayberg et al., 2005). Before DBS, patients compared to HCs showed higher negative versus positive affective sharing which was associated with enhanced beta-band desynchronization in the sgACC. This desynchronization correlated with self-reported severity of depressive symptoms. After 6 months of DBS, patients showed normalized empathic responses in the sense of no differences between empathic involvement ratings for persons who depicted negative emotions versus for those who depicted positive emotions. As neural oscillations are a crucial mechanism for coordinated neural functioning (Buzsáki and Draguhn, 2004) and as DBS has been shown to successfully suppress disruptive pathological oscillatory activity (Eusebio et al., 2011), these findings might be reflective of sgACC DBS improving clinical symptoms in MDD. Considering that the reduction of empathic affect sharing with negative stimuli showed a tendency to correlate with symptom improvement, these findings further support the notion of a mental-health-promoting effect of reduced empathic involvement with negative emotions in MDD.

In a similar vein, Kilian et al. (2024) applied DBS to the supero-lateral medial forebrain bundle (slMFB) in patients with treatment-resistant MDD and HCs. As the slMFB shows overlaps with neural correlates of socio-affective and -cognitive capacities and, as a connecting structure of the mesolimbic pathway, induces brain metabolism changes not only in the stimulated area but also distal to it, authors have pointed out the plausibility of slMFB DBS to modulate social affect. Before and 3 months after DBS, participants performed a task in which they were presented with naturalistic video stimuli of narrators presenting allegedly autobiographic stories that were either emotional or neutral in valence. Based on subjective ratings of affective valence, compassion, and answers to a multiple-choice question on the content of the video, not only affective empathy, but also related constructs of cognitive empathy and compassion were enquired (Kanske et al., 2015, 2016). While before DBS, affective empathy significantly differed between HCs and patients with MDD, these differences were not evident after 3 months of DBS. This effect was driven by changes from baseline to follow-up in the MDD group: Specifically, a reduced negativity bias in their affect ratings after watching neutrally valenced stimuli was observed. The supposedly normalized affective responsiveness in patients was accompanied by lower levels of self-reported depressive symptoms. No treatment effects occurred regarding persistingly lower levels of compassion and intact socio-cognitive skills in patients versus HCs both pre- and post-DBS.

**Figure 1:**
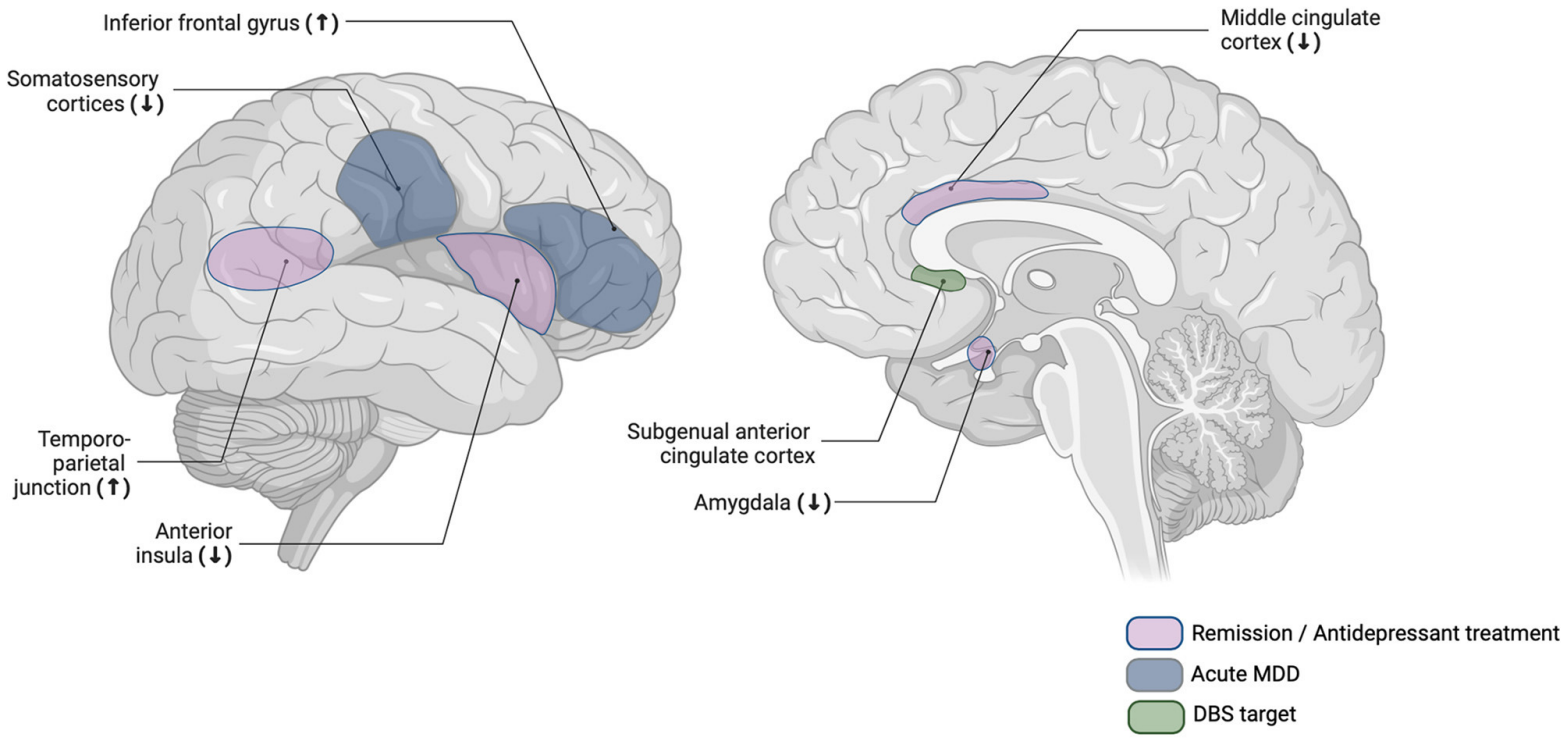
Schematic overview of brain regions that have been reported to show aberrant activity in acute MDD or after remission or antidepressant treatment. DBS, deep brain stimulation; MDD, major depressive disorder.

Pointing out the anatomical and functional coupling between the slMFB and the sgACC, the authors suggest their findings together with Merkl et al.'s (2016) findings to show a network-specific effect of DBS in MDD. The suggested relevance of DBS-induced normalized affective responsiveness for long-term antidepressant effects is to be further investigated, as well as preliminary indications in Kilian et al.'s (2024) study on the specific malleability of affective empathy compared to other socio-affective and -cognitive functions. Importantly, these DBS studies do not only support the notion of reduced empathic responding to contribute to symptom improvement in MDD but moreover allow for a more detailed insight into the specific nature of aberrations revealing MDD-related negativity biases in empathic responding (see Figure 1 for overview of reviewed findings).

## 4 A proposed model of associational shifts between empathy and MDD throughout the course of the disorder

While the studies' heterogeneity renders the aggregation of findings challenging, it also provides important insights insights into a complex and potentially state-dependent association between MDD and empathy. Although preliminary, we suggest the neural activity findings to reflect a differential association between empathy and MDD depending on the state of the disorder and treatment effects. While elevated neural activity in empathy-related brain areas during acute symptomatology might reflect elevated empathic distress (Fujino et al., 2014)—also in accordance with findings on elevated self-reported empathy in MDD (Schreiter et al., 2013)—antidepressant treatment or remission might dampen empathic reactions toward negative stimuli. This notion is reflected in lower activity in empathy-related brain areas such as the AI and MCC after remission or antidepressant treatment compared to pretreatment or acute episodes, and is further supported in lower self-reports of affective empathy (Merkl et al., 2016; Rütgen et al., 2019; Kilian et al., 2024).

Conceivably, this associational shift might reflect a coping mechanism in the aftermath of elevated empathy in MDD, fostering compensatory, more detached empathic responding. The finding of elevated TPJ activity in patients with remitted compared to acute MDD and HCs could potentially indicate a mechanism enabling this dampened empathy: As the TPJ has been associated with higher self-other distinction and more cognitive aspects of empathy (Schurz et al., 2021), an elevated cognitive processing of others' negative emotions might enable individuals to be less overwhelmed by another's negative emotion and thereby counteract the MDD-related proneness to show empathic distress. It, thus, acts as a possible way to regulate difficult emotions, which bears importance given the described emotion regulation deficits in MDD (Kanske et al., 2012; Joormann and Quinn, 2014).

**Figure 2:**
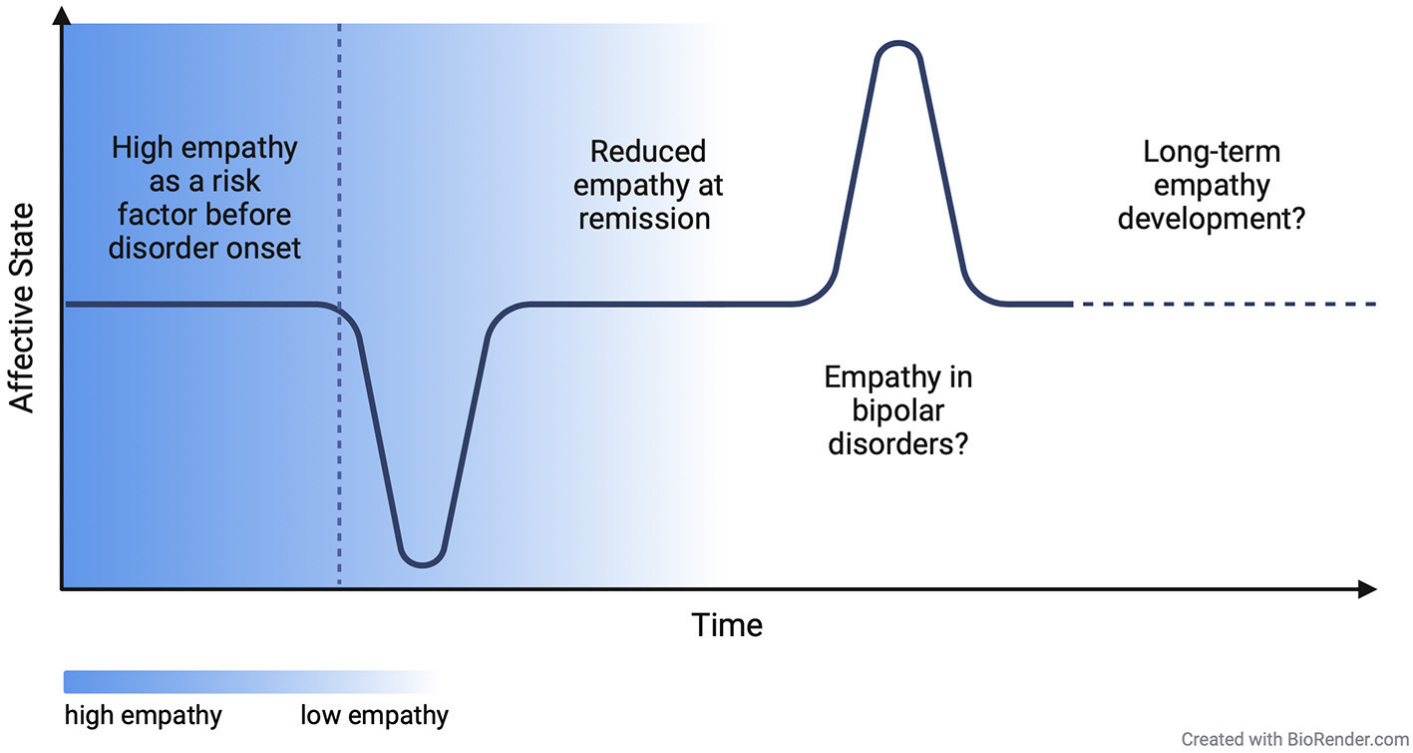
Proposed model of associational shift between empathy and affective symptoms throughout the course of the disorder.

This suggested associational shift however requires further investigation in future longitudinal studies. Moreover, longer term implications of dampened empathic responding should be investigated, potentially yielding the risk of lower opportunities for rewarding social interactions in the long run (Trew, 2011; see Figure 2 for schematic depiction of proposed model).

## 5 Outlook

Since empathic responding does not evolve in a vacuum but is most probably significantly influenced in its emergence and outcomes depending on a specific composition of various moderators, future studies should take those moderators into consideration. Further affective and cognitive mechanisms that might come into play in real life social interactions and should be amongst others comprise cognitive empathy, emotion regulation (Tully et al., 2016; Guendelman et al., 2022), or executive functioning (Thoma et al., 2011).

Particularly neural correlates of cognitive empathy should be integrated in future studies due to their potentially interacting effects with affective empathy on the emergence of MDD. Importantly, a well-regulated interplay between cognitive and affective mechanisms has been suggested to show the most adaptive outcomes by allowing for understanding another's emotions and being affectively involved without becoming overwhelmed (Tully et al., 2016; Calandri et al., 2019). Since empathy can be constrained by information-processing biases channeling certain environmental input, cognitive empathy could be crucial to reduce these biases (Decety, 2021). Building upon previous findings on cross-network interactions during socio-affective and -cognitive processing (Schurz et al., 2020), research on potential changes in these interactions throughout the course of MDD could be of particular relevance.

Lastly, in order to elucidate the time-wise dynamics in the association between empathy and MDD, future studies should apply longitudinal approaches, that furthermore not only include patients with acute MDD and remitted MDD but also at-risk individuals before disorder onset. Based on this study design, it is to be investigated whether altered empathy is the antecedent or the consequence of MDD: While aberrations in empathy might be a vulnerability factor before the onset of MDD, depressive symptoms may heighten the emotional sensitivity toward others' emotions and alter an individual's empathic responding even after remission (Schreiter et al., 2013). Interestingly, Ding et al. (2023) have provided first longitudinal evidence on bidirectional positive associations between of empathy and depressive symptoms. Since their analyses were based on self-reports of empathy only, further research including neural activity data might yield relevant insights into the underlying mechanisms. Taken together, we suggest future studies to provide multimodal, longitudinal designs on stratified samples, ideally comprising affective as well as cognitive measures of empathy.

## 6 Clinical implications

From a clinical point of view, our findings add to previous indications of the efficacy of reversing the negativity bias in antidepressant treatment (Harmer et al., 2009; Rottenberg and Hindash, 2015) and extend its relevance to respective biases in affective empathy. It is still to be investigated whether this bias can be replicated for MDD specifically, or whether it might show the potential as a transdiagnostic marker of aberrant social affect for various mental disorders. Considering previously reported mood-congruent processing biases (Leppänen, 2006; Sterzer et al., 2011), extending investigations to potentially diverging biases in bipolar disorders might contribute to our clinical understanding of those mental disorders.

On a more general note, the consistency in results on elevated empathy to be associated with depressive symptoms highlight the relevance of adopting a critical perspective on the mental health impacts of fostering socio-affective and -cognitive capacities. Emerging indications of moderate levels of empathy to show the most adaptive outcomes (Tully et al., 2016) should find consideration in our notion of desirable social affect and cognition in social interactions and accordingly adjusted psychoeducation in clinical settings.

## 7 Conclusion

In this review, we have shed light on the complex relationship between MDD and neural correlates of affective empathy. Patients with MDD compared to HCs show seemingly state-dependent neural aberrations during empathic processing. Predominately post-treatment or after remission, lower empathy-related activity has been found in brain regions such as the AI, IFG, and cingulate areas, partly accompanied by reductions in subjective negative empathic affect sharing. Further longitudinal and multimodal research is required to test the suggested state-dependent associational shift between empathy and MDD. Prospectively, this might provide a more informative basis for psychotherapeutical interventions targeting aberrant social affect in various psychopathological conditions.

## Author contributions

DC: Conceptualization, Writing – original draft. KF: Writing – review & editing. NA: Writing – review & editing. PK: Supervision, Writing – review & editing.
